# Dual trigger with gonadotropin-releasing hormone and human chorionic gonadotropin for poor responders

**DOI:** 10.4274/jtgga.2017.0045

**Published:** 2018-06-04

**Authors:** Ahmet Eser, Belgin Devranoğlu, Evrim Bostancı Ergen, Ciğdem Yayla Abide

**Affiliations:** 1 Department of Gynecology Obstetrics and Reproductive Medicine, İstanbul Zeynep Kamil Woman and Child Diseases Training and Research Hospital, İstanbul, Turkey

**Keywords:** Dual trigger, intracytoplasmic sperm injection, poor responders

## Abstract

**Objective:**

To compare metaphase II (MII) rate, fertilization rate, and embryo quality with dual trigger gonadotropin-releasing hormone agonist (GnRH) and normal dose human chorionic gonadotropin (hCG) versus a normal dose hCG trigger in antagonist intracytoplasmic sperm injection (ICSI) cycles of poor ovarian responders.

**Material and Methods:**

Patients defined as poor ovarian responders according to the Bologna criteria who underwent ICSI with GnRH antagonist protocol and triggered with dual trigger or hCG alone for oocyte maturation. Main outcome measures were MII rate, fertilization rate, and embryo quality.

**Results:**

Total gonadotropin doses and E_2_ levels on trigger day were higher in the hCG trigger group. There were no significant differences with regard to implantation rate (p=0.304), biochemical pregnancy rate (p=0.815), clinical pregnancy rate (p=0.378), and ongoing pregnancy rate (p=0.635) between the groups.

**Conclusion:**

Dual trigger of oocyte maturation with GnRH agonist and normal dose hCG in poor responders does not demonstrate improved oocyte maturation, clinical pregnancy, and ongoing pregnancy rates.

## Introduction

Poor ovarian response (POR) is known as the decrease of fecundity. The first systematic definition of POR was identified as the Bologna criteria in 2011 by The European Society of Human Reproduction and Embryology (1). Patients with POR have very poor outcomes despite improving treatment modalities such as the use of different stimulation protocols or adding adjuvant therapies (2).

Human chorionic gonadotropin (hCG) is almost always used to trigger final oocyte maturation and is required to pick-up mature oocytes from stimulated ovaries in in vitro fertilization (IVF) and intracytoplasmic sperm injection (ICSI) cycles. After the administration of gonadotropin-releasing hormone agonist (GnRH) antagonists in IVF/ICSI cycles, triggering with GnRH agonists and other methods (double trigger and dual trigger) has been introduced (3,4,5,6). These triggering methods provide the release of endogenous follicle-stimulating hormone (FSH) and luteinizing hormone (LH) surge like in the natural cycle for the maturation of follicles. “Dual trigger” was first introduced by Shapiro et al. (6) in co-treated patients with GnRH antagonist cycles for the purpose of Ovarian Hyperstimulation syndrome (OHSS) prevention. Besides prevention of OHSS, Lin et al. (7) demonstrated improved implantation, clinical pregnancy, and live-birth rates in normal responders using the dual trigger regimen.

Even though benefits were shown when using the dual triggering regimen in high-responder and normal-responder patients or having oocyte immaturity, few studies have been performed to show the effects when using this regimen in the poor-responder population (7,8,9,10,11,12,13,14).

The aim of the present study was to analyze whether the dual trigger in POR might improve ICSI cycle outcomes.

## Material and Methods

This case-control study was approved by the institutional ethics committee. A total of 47 ICSI cycles in which a dual trigger was used for final oocyte maturation were performed from March 2015 until July 2015. Moreover, a review of medical records from May 2012 through April 2014 was performed for poor responders who were triggered with hCG. Controls included 62 ICSI cycles. Both cases and controls were recruited consecutively.

### Participants and treatment protocol

All patients who fulfilled the criteria defined by the European Society of Human Reproduction and Embryology consensus in 2011 and underwent ICSI cycles with GnRH antagonist were considered eligible (1). The Bologna criteria for poor responders was defined as the presence of two of the following features: 1) Increased maternal age (40 years) or other risk factors for POR, 2) A previously demonstrated POR (≤3 oocytes with a conventional stimulation protocol), 3) An abnormal ovarian reserve test results (i.e., antral follicular count <5-7 follicles or anti-Müllerian hormone <0.5-1.1 ng/mL) (1). Patients with other infertility factors were excluded from the study. 

Patients underwent controlled ovarian hyperstimulation with the multi-dose GnRH antagonist and with a starting gonadotropin (recombinant FSH or human menopausal gonadotropin) dose of ≥300 IU, which were administered from the second day of the cycle. GnRH antagonist (Ganirelix; Merck Sharp and Dohme) was started 0.25 mg subcutaneously from the day that the diameter of the leading follicle reached ≥14 mm or serum estradiol (E_2_) reached >350 pg/mL, until the day of the trigger. Patients were excluded from the study whose cycles were cancelled because of unresponsiveness to the gonadotropins. Cases were triggered with a combination 250 mcg choriogonadotropin alpha (Ovitrelle; Merck) plus 0.2 mg triptoreline acetate (Gonapeptyl; Ferring) subcutaneously when follicles reached ≥17 mm in diameter. Controls were triggered only with 250 mcg choriogonadotropin alpha when follicles reached ≥17 mm in diameter. Serum E_2_ levels were assessed on the day of the trigger. Transvaginal ultrasound-guided oocyte picks up was performed 35 hours after triggering.

Luteal phase supplementation was provided by daily administration of 90 mg vaginal progesterone gel (Crinone; Serono) from the day after oocyte pick up until either a negative pregnancy test or 10 weeks of gestation. If patients had embryos after oocyte retrieval, transfer day and the number of transferrable embryos were assessed according to embryo quality and number of embryos. One proficient physician transferred whole embryos that were at the cleavage stage. Embryo quality was based on cleavage and morphology scores assessing the size equality and percentage of the fragmentation rate of the cells, as described by Veeck (15). 

Serum β-hCG level was measured 14 days after embryo transfer and positive pregnancy was defined above the level of >5 IU/L.

### Outcome variables

The primary outcome was MII, fertilization and top-quality embryo rates. Secondary outcomes were clinical and ongoing pregnancy rate. Clinical pregnancy was defined as the presence of a positive heart beat after 4 weeks of positive pregnancy.

### Statistical analysis

Statistical analyses were performed using the NCSS (Number Cruncher Statistical System) 2007 (Kaysville, Utah, USA). Data are presented as mean, standard deviation, median (range), ratio, minimum and maximum. Student’s t-test was used to compare parametric data and the Mann-Whitney U test was used to compare non-parametric data. Qualitative clinical outcomes were examined using Fisher’s exact test. P<0.05 was considered statistically significant.

## Results

The baseline characteristics are shown in Table 1. In the hCG trigger group, day 3 FSH (9.6±5 vs 11±3.7, p=0.006) was significantly higher than the dual trigger group. Other characteristics did not significantly differ between the dual trigger group and the hCG trigger group.

In terms of the cycle characteristics of the two groups, total dose of gonadotropins (3165.4±1124.2 vs 3839.5±805.5 IU, p=0.001) and E_2_ on trigger day (647.5±361.9 vs 923.9±603.1 pg/mL; p=0.017) were significantly higher in the hCG trigger group. Other parameters did not significantly differ between the dual trigger group and the hCG trigger group (Table 2).

Dual trigger group compared with hCG trigger group with regards to implantation rate, biochemical pregnancy rate, clinical pregnancy rate, and ongoing pregnancy rate. There was no statistically significant differences between ICSI outcomes (Table 3). We detected no OHSS in either group.

## Discussion

This case-control study assessed the effect of dual triggering through an antagonist stimulation protocol in poor responder women undergoing ICSI cycles. Although total gonadotropin doses and E_2_ levels on the trigger day were higher in hCG trigger group, the results of this study suggest there was no clinical difference when a dual trigger was used instead of an hCG trigger in poor responder women.

hCG triggers are used conventionally in IVF/ICSI cycles. Although this technique is thought to be successful, researchers are investigating new tools to prevent OHSS and to improve the extent of mature oocytes obtained. 

The GnRH agonist trigger was first introduced by Gonen et al. (3). Triggering with a GnRH agonist causes the release of both FSH and LH a natural cycle flares up, which is considered to be more physiologic. A Cochrane meta-analysis showed triggering with GnRH agonist instead of hCG was an acceptable method by transferring freeze/thaw embryos compared with conventional trigger in fresh cycles. The GnRH agonist trigger had similar live birth rates with a substantial reduction in OHSS rates (16). After an agonist trigger was defined, triggering with hCG and FSH concomitantly showed the improvement of oocyte maturation and fertilization in a previous study (17). GnRH receptors were identified in the endometrium, in preimplanted embryo, and in ovarian granulosa cells other than in the pituitary, and ovulation has been regulated by GnRH (18,19,20). Moreover, Raga et al. (21) showed that a GnRH agonist improved preimplantation embryonic developments in a murine model, independent of FSH.

The dual trigger was first introduced by Shapiro et al. (6). Despite there being a scarcity of studies that investigated the impact of a dual trigger in the literature; a dual trigger with standard dose hCG provided higher oocyte retrieval numbers (7,12), higher numbers of retrieved M2 oocytes (7,12), higher M2 oocyte rates (7,22), higher numbers of cryopreserved embryos (7,23); and improved implantation (7), clinical pregnancy (7,14) and live birth rates (7) in normal responder patients.

In our study, we hypothesized that a GnRH agonist and release of FSH due to a GnRH agonist flare up might have dual influence and may enhance oocyte quality, M2 oocyte rate, fertilized oocytes, and embryo quality, without affecting endometrial receptivity and implantation in poor responders. However, we found no differences in M2 oocyte retrieval, M2 oocyte rates, number of total oocytes retrieved, fertilized oocytes, fertilization rates, top and good quality embryos, and top-quality embryo rates between the dual trigger group and the hCG trigger group. Despite an enhancement of IVF/ICSI outcome when triggering with dual triggers in normal responder patients, the lack of difference between these parameters may depend on the reason of an underlying oocyte dysfunction. Aneuploidy and poor oocyte maturity is still the main problem needed to be solved in poor responder patients (24).

Although GnRH agonist triggers have been shown to induce oocyte maturation, low pregnancy and increasing miscarriage rates were associated with luteal phase insufficiency (25,26). Intensive luteal E_2_ and progesterone were used to provide luteal phase support but results were conflicting. Babayof et al. (27) used this protocol first in patients at high risk for OHSS and this study showed poor reproductive outcomes. This result may be due to their low number of patients. In a previous prospective randomized study, Engmann et al. (28) found similar implantation and clinical pregnancy rates in patients at high risk for OHSS undergoing IVF. Despite using intensive luteal support, a retrospective study showed decreased implantation and pregnancy rates in OHSS patients with high risk (29). Other methods to support the luteal phase with GnRH agonist trigger include the use of hCG after the GnRH agonist trigger and dual trigger. Modified luteal phase support with hCG after a GnRH agonist trigger has shown similar results in implantation, and clinical and ongoing pregnancy rates compared with hCG (30).

In the dual trigger method, hCG prevents the luteolytic effect of GnRH agonist and provides adequate luteal phase support (6). Although the implantation and pregnancy rates were not significantly different in normal responder patients undergoing ICSI in a previous randomized controlled study, other reports showed promising pregnancy results (7,8,14,23). In our study, implantation and clinical and ongoing pregnancy rates were not statistically significant. According to the previously mentioned studies, luteal phase deficiency was not a concern in dual trigger cycles. However, the lack of difference between implantation, clinical and ongoing pregnancy rates in our study might be due to our patient population who were all poor responders.

A limitation of this study is that it was underpowered regarding the implantation rate, biochemical pregnancy rate, clinical pregnancy rate, and ongoing pregnancy rate due to the low poor responder population in our clinic. Another limitation is that the hCG trigger group was recruited retrospectively.

In conclusion, no statistical significance was found between a dual trigger and conventional hCG trigger. However, larger prospective randomized controlled studies are needed to evaluate whether a dual trigger enhances oocyte maturation and improves ICSI outcome in poor responders.

## Figures and Tables

**Table 1:**
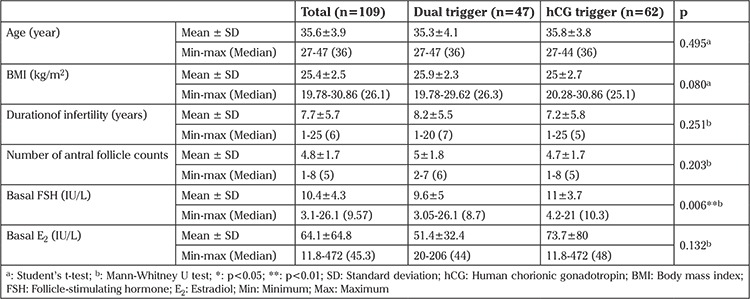
Comparison of the dual trigger and human chorionic gonadotropin trigger: demographic characteristics

**Table 2:**
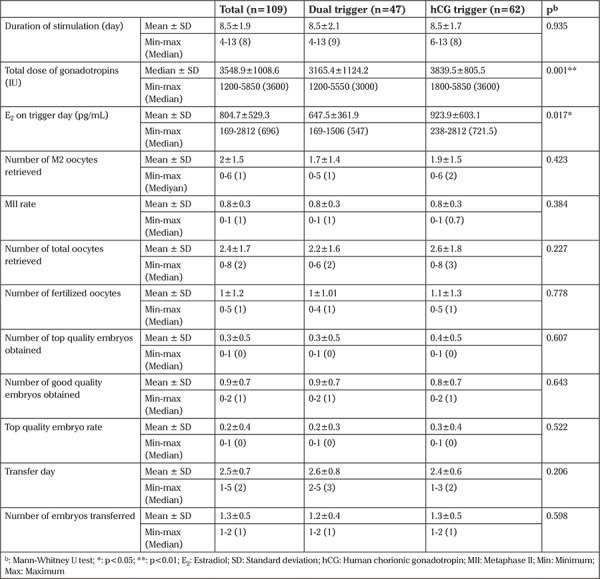
Comparison of the cycle characteristics between the dual trigger and human chorionic gonadotropin trigger

**Table 3:**
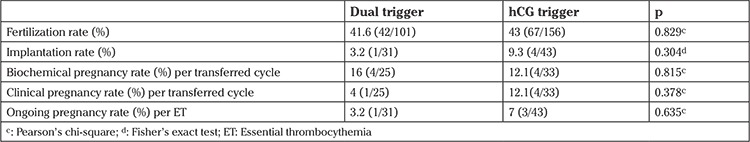
Comparison of the standard and dual trigger methods
